# The central inflammatory regulator IκBζ: induction, regulation and physiological functions

**DOI:** 10.3389/fimmu.2023.1188253

**Published:** 2023-06-12

**Authors:** Yanpeng Feng, Zhiyuan Chen, Yi Xu, Yuxuan Han, Xiujuan Jia, Zixuan Wang, Nannan Zhang, Wenjing Lv

**Affiliations:** ^1^ Department of Neurosurgery & Pathophysiology, Institute of Neuroregeneration & Neurorehabilitation, Qingdao University, Qingdao, China; ^2^ Department of Geriatrics, The Affiliated Hospital of Qingdao University, Qingdao, China

**Keywords:** IκBζ, inflammation, tumor, psoriasis, toll-like receptors, mRNA

## Abstract

IκBζ (encoded by NFKBIZ) is the most recently identified IkappaB family protein. As an atypical member of the IkappaB protein family, NFKBIZ has been the focus of recent studies because of its role in inflammation. Specifically, it is a key gene in the regulation of a variety of inflammatory factors in the NF-KB pathway, thereby affecting the progression of related diseases. In recent years, investigations into NFKBIZ have led to greater understanding of this gene. In this review, we summarize the induction of NFKBIZ and then elucidate its transcription, translation, molecular mechanism and physiological function. Finally, the roles played by NFKBIZ in psoriasis, cancer, kidney injury, autoimmune diseases and other diseases are described. NFKBIZ functions are universal and bidirectional, and therefore, this gene may exert a great influence on the regulation of inflammation and inflammation-related diseases.

## 1 Introduction

IκBζ is a key inflammatory factor belonging to the IkappaB protein family that plays a central role in the expression of cytokines, regulating the functions of Mononuclear phagocyte system, NK cells, T cells and B cells. An increasing number of publications are showing that IκBζ is related to autoimmune diseases and regulates the release of pro-inflammatory or anti-inflammatory factors through the NF-κB pathway to cause autoimmune diseases and inhibit or promote tumors. In this review, we mainly summarize the role and mechanism of IκBζ in the regulation of various cytokines, autoimmune diseases tumorigenesis and development.

## 2 IkappaB protein families

**Figure 1:**
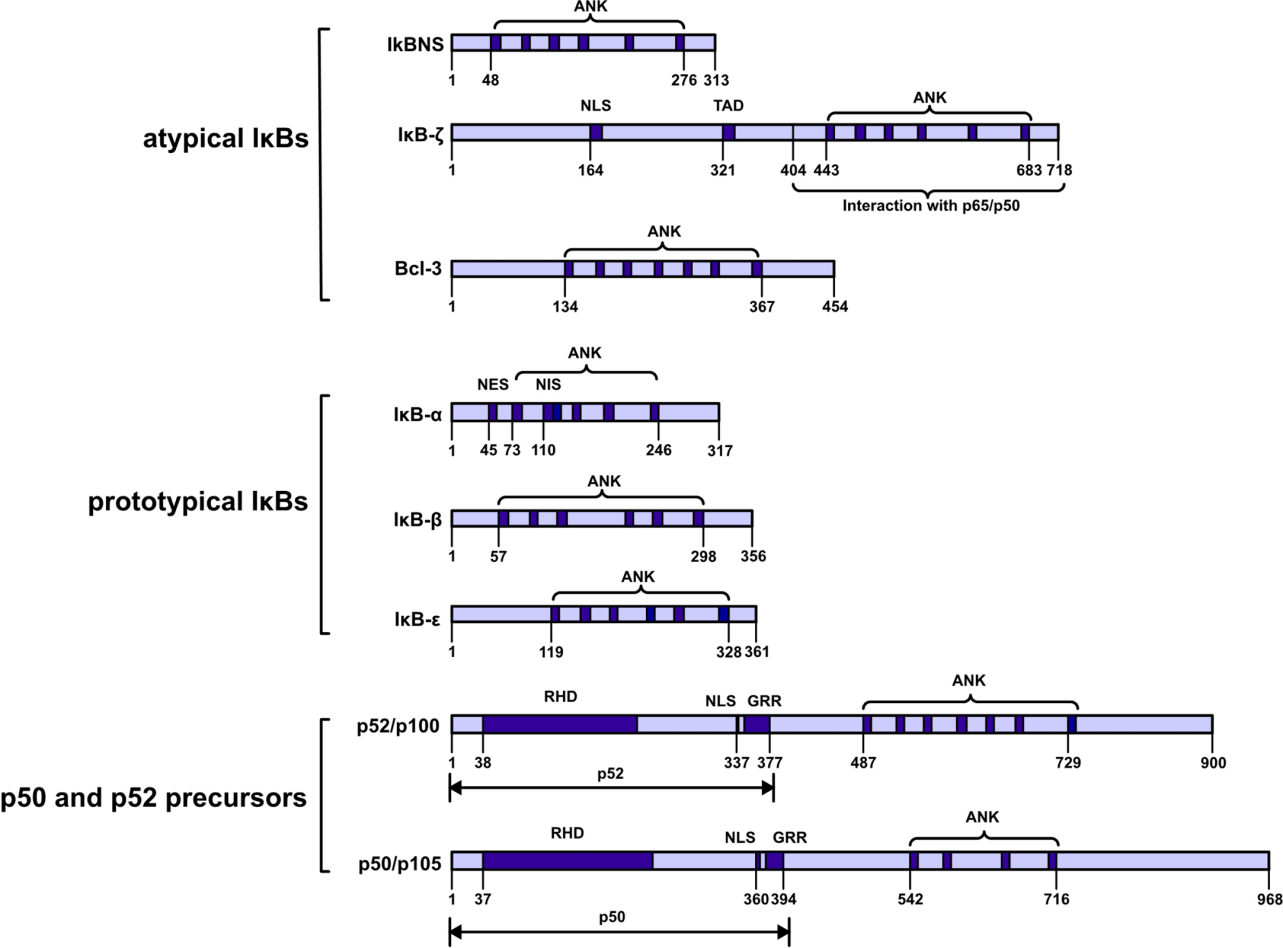
Schematic diagram showing the IkappaB protein families. IκBs can be further classified into three subfamilies: prototypical IκBs, atypical IκBs, and p50 and p52 precursors. Atypical IκBs localize to the nucleus, while the other subfamily proteins localize to the cytoplasm. Ankyrin-repeat domain (ANK); nuclear localization signal (NLS); transactivation domain (TAD); nuclear export signal (NES); nuclear import signal (NIS); REL homology domain (RHD); glycine-rich region (GRR); death domain (DD).

These proteins can be further classified into three subfamilies: prototypical IκBs (IκBα, IκBβ, and IκBϵ); atypical IκBs (Bcl-3, IκBNS and IκBζ); and the p50 and p52 precursors p105 and p100. These proteins are also categorized into two groups on the basis of their localization either in the cytoplasm (IκBα, IκBβ, IκBϵ, p100 and p105) or nucleus (Bcl-3, IκBNS and IκBζ). All seven members share a common feature: ankyrin‐repeats (ANKs) in the COOH‐terminal region, and these repeats are believed to inhibit the activities of the NH_2_ -terminal region *via* molecular mechanisms. Notably, ANKs cover the nuclear localization sequence (NLS) in the Rel homologous domain of NF-κB to inhibit NF-κB activity (1) (**Figure 1**).

The cytoplasmic IκB protein binds to the rel homologous domain of the NF-KB subunit through the subunit ANK sequence and thus inhibits NF-KB nuclear localization. After a stimulus triggers the activation of the IκB kinase (IKK) complex, the serine near the amino end of the IκB protein is phosphorylated, and then, the IκB protein is ubiquitinated and ultimately degraded by the proteasome. Thus, NF-KB can be translocated into the nucleus and bind to target genes. Cytokine receptors such as the TNF receptor and IL-1 receptor; pattern recognition receptors such as TLR4; and many other stimuli activate signaling cascades that culminate with the activation of IKKβ after identification of the ligand. IKKβ belongs to a complex that also includes the regulatory protein IKKγ and the related kinase IKKα. The NF-κB signaling pathway is classified into canonical, noncanonical, and atypical signaling pathways. IKKα is both a dependent and an independent factor in the noncanonical route. Cytokines activate IKKα, which in turn causes p100 phosphorylation and the formation of p52/RelB complexes. IKKα contributes to certain canonical signaling pathways, and activation of these canonical pathways enhances noncanonical signaling through the induction of p100 expression (2, 3).

Nuclear IκB proteins are hypothesized to control the transcriptional activity of NF-κB. IκBζ has a TAD that can interact with transcription factors, histone and regulatory protein to promote the initiation of gene transcription BCL-3 and IκBNS have no tad, so they have no transcriptional activity (4, 5). IκBζ prevents p65/p50 heterodimers and p50/p50 homodimers from binding DNA (6). By controlling NF-κB transcriptional activity or its stability on DNA, BCL-3 functions as either an activator or an inhibitor of NF-κB in a context-specific manner (7). IκBNS interacts with DNA-bound p50 homodimers and prevents NF-κB dimers from attaching to promoters (8).

## 3 Structure of IκBζ

**Figure 2:**
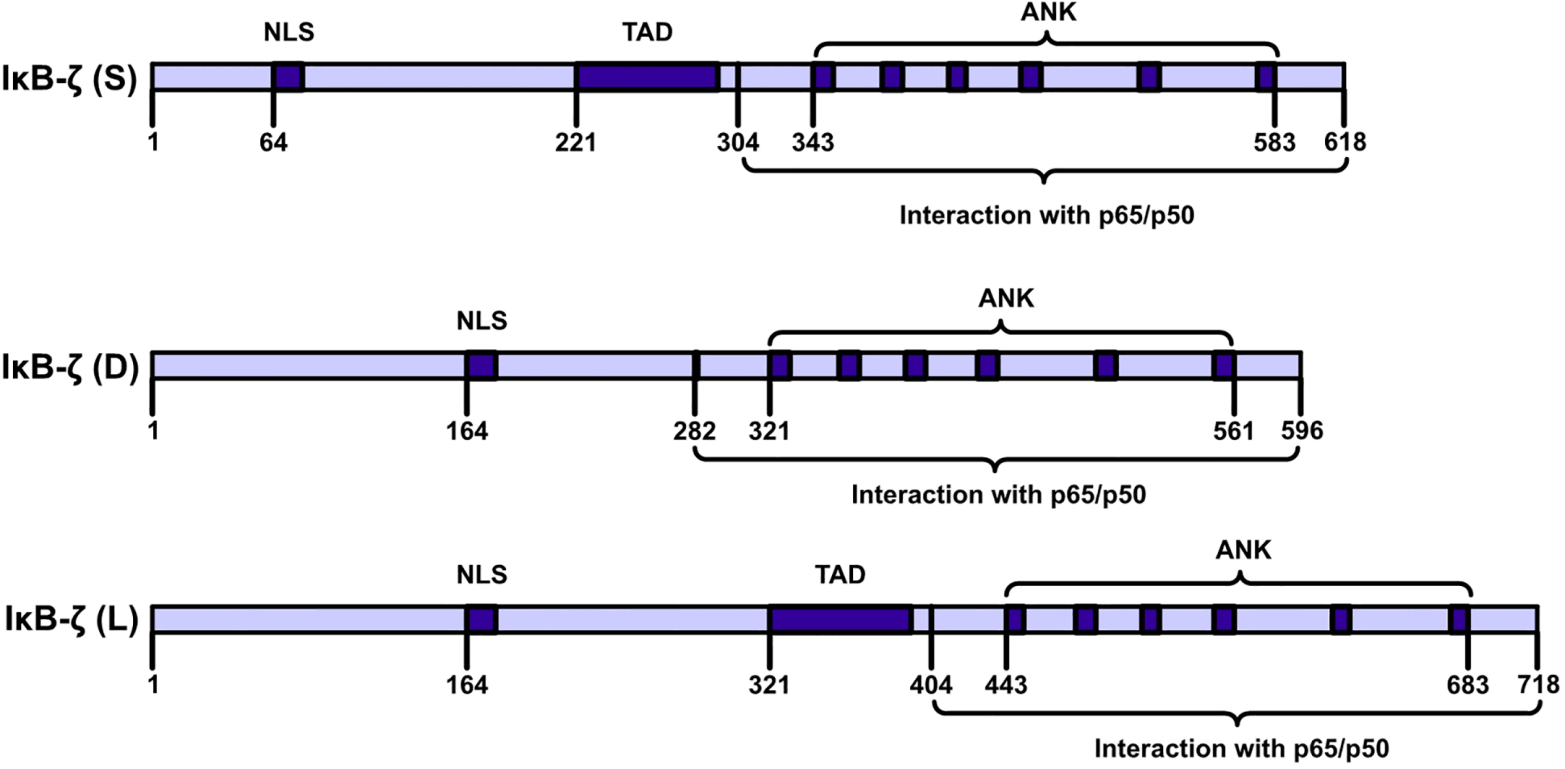
IκBζ isoforms NFKBIZ produces three. IκBζ isoforms. The longest isoform, IκB-ζ(L), comprises 728 amino acids (AAs); the shortest isoform, IκB-ζ(S), comprises 629 AAs; and the smallest isoform, IκB-ζ(D), carries 534 AAs. IκB-ζ(L) and IκB-ζ(S) harbor TADs, NLSs, and ANKs, and these isoforms engage in auto- and transactivation and inhibit NF-κB action. IκB-ζ(D) lacks AAs 236-429, and IκB-ζ(L) lacks transactivation activity and inhibits the activity of NF-κB.

IκBζ is a member of the IkappaB family of proteins and carries ANK repeats (9, 10). IκBζ is also called interleukin (IL)-1-inducible nuclear ankyrin-repeat protein (INAP) or a molecule possessing ankyrin‐repeats induced by lipopolysaccharide (MAIL). (11–13). The C-terminal half of the full sequence (amino acids [AAs], 453–718) carries six typical ANKs, which are clustered in the sequences of IkappaB protein family members (11). IκBζ is encoded by NFKBIZ (NFKB inhibitor zeta), a gene with 14 exons (14). The NFKBIZ gene is expressed as seven transcript-spliced variants, which are categorized into three subtypes: IκBζ: IκBζ(L), IκBζ(S) and IκBζ(D). IκBζ(L) and IκBζ(S) exist in cells. IκBζ(L) and IκBζ(S) are the two functional subtypes in this category. The IκBζ(L) transcript carries an open reading frame comprising 2187 bp and encodes a polypeptide comprising 718 AAs. IκBζ(L) is encoded by all 14 exons in NFKBIZ. IκBζ(S) is encoded by NFKBIZ lacking the third exon, and an artificial form of IκBζ(D) was artificially designed by Prof. Muta and it is encoded by NFKBIZ lacking the seventh exon (**Figure 2**). The major expression product of NFKBIZ is IκBζ(L) (10, 15). IκBζ(L) was found at high levels in IL-1β-treated mouse chondrocytes and in human monocyte cells after lipopolysaccharide (LPS) treatment and rIL-1β induction after 4 h (16, 17). IκBζ (L) exerted more robust physiological effects than exerted by IκBζ (S) (18). IκBζ (D) exerted no physiological effect. An analysis of DNase I and Pol II ChIP-sequencing (ChIP-seq) data showed that IκBζ (L) and IκBζ (S) not only are produced by alternative splicing but are also driven by two different promoter regions and carry different transcription initiation sites (19).

## 4 NFKBIZ stability

### 4.1 NFKBIZ mRNA stability depends on the 3′-UTR structure and the IL1-R/TLR signaling

**Figure 3:**
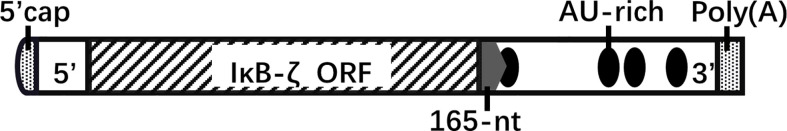
NFKBIZ mRNA structure. The 3’-UTR of NFKBIZ mRNA, which lacks protein-coding capability, harbors sequences that play a role in translational regulation. Specifically, the 165-nucleotide sequence located at the beginning of the 3’-UTR influences the stability of IκBζ. Within this sequence, a cis-element with specific inducible effects is present. Additionally, the 3’-UTR of NFKBIZ mRNA contains four AU-rich elements that contribute to mRNA stability. Notably, the first AU-rich element is partially located within the 165-nucleotide region. five prime cap(5’-cap); open reading frame(ORF); AU-rich element(ARE); polyadenosinic acid(poly(A)).

A 165-nucleotide sequence in the 3′-untranslated region (UTR) of IκBζ mRNA influences the mRNA stabilization of IκBζ; it contains the cis-element involved in stimulus-specific induction (**Figure 3**). Stimulation by il-1βor fusion mRNAs carrying the full-length IκBζ mRNA or 165-nucleotide fragment exhibited similar activity (20). The RNA-binding protein AT-rich interactive domain-containing protein 5a (Arid5a) binds to the 3’-UTR of IκBζ mRNA and promotes the translation of IκBζ (21). AU-rich elements are usually associated with altered mRNA stability, particularly in the 3’-UTR. However, the AU-rich elements of NFKBIZ (AUUUA) are dispensable for the stability of IκBζ mRNA (22).

MicroRNAs (miRNAs) also affect mRNA stability by recognizing and then forming base pairs with sequences in the 3’-UTR (23). This is a way to influence the inflammatory function of NFKBIZ. miR-4734, miR-376b, miR-124a and miR-187 inhibit the expression of NFKBIZ and target a sequence in the 3′-UTR of *NFKBIZ* mRNA. miR-4734 suppresses Endoplasmic Reticulum stress-associated proinflammatory responses by affecting NKFBIZ mRNA stability. miRNA targets (miR-484; miR-374, miR-410, and miR-369-3p; miR-376) are dispensable for the stability of IκBζ mRNA (22, 24) (**Table 1**).

**Table 1:** Some microRNA that directly target NFKBIZ.

MicroRNA	Cells	Disease model	Function	Target Sequence(5’-3’)	References
miR-376b	renal tubular cells in mice with septic AKI(acute kidney injury)/Hepa1-6 cells	septic acute kidney injury /partial hepatectomy	suppressed the expression of IκB-ζ	UCUAUGAA	(25, 26)
miR-124a	HepG2 cells	/	suppressed the expression of IκB-ζ	UUGCCUUA	(27)
miR-187	HEK293 cells/macrophages cells	/	suppressed the expression of IκB-ζ	AGACACG	(28, 29)
miR-484	RAW264.7 cells	/	dispensable	GAGCCUGG	(22)
miR-374, miR-410, miR-369-3p	RAW264.7 cells	/	dispensable	UCUAUGAA	(22)
miR-376	RAW264.7 cells	/	dispensable	UAUUAUAU	(22)

The stability of IκBζ mRNA is affected by the upstream signaling molecules that induce IκBζ production. Stimulation of IL-17 stabilizes IκBζ mRNA and significantly prolongs the NFKBIZ half-life (3, 30, 31). In the absence of IL-1β stimulation, IκBζ mRNA exhibits significantly reduced activity (20). The mRNA stabilization of IκBζ depends on the recruitment of IRAK1 to the TIR domains in IL-1R and TLR receptors (32). MyD88 plays a crucial role in the stabilization of IκBζ mRNA. The half-life of IκBζ mRNA is reduced in MyD88-deficient macrophages (22). The monocyte chemotactic protein-inducing protein (MCPIP) family is a CCCH zinc finger protein family (33). MCPIP1 is an endoribonuclease that inhibits TLR signaling by degrading mRNA transcripts through the 3’-UTR stem−loop motif (34). MCPIP1 degrades NFKBIZ mRNA and impairs the activation of specific promoters, such as the il-6, lcn2, Il17ra and Il17rc promoters (35). TANK-binding kinase 1 (TBK1) is required for IκBζ mRNA stabilization mediated *via* MCPIP1 phosphorylation (36). MCPIP3 can directly degrade IκBζ mRNA to regulate the NF-κB pathway (37, 38).

### 4.2 NFKBIZ posttranslational regulation depends on the redox environment and its phosphorylation

IκBζ is a redox-sensitive protein; reactive oxygen species (ROS)-dependent regulation is one of the mechanisms by which IκBζ protein is stabilized (39). Quinone oxidoreductase 1 (NQO1), an antioxidation stress protein, is a scaffold that facilitates the association between IκBζ and PDLIM2 (a nuclear ubiquitin E3 ligase); then, IκBζ is ubiquitinated and is ultimately degraded by the proteasome (40). IL-20 promotes IκBζ degradation in hepatocytes *via* the induction of NQO1. IκBζ protein was upregulated in the liver in IL20^−/−^ mice, but IκBζ mRNA expression was comparable to that in WT mice, suggesting that IL-20 reduced the stability of the IκBζ protein without affecting mRNA expression (41). Activators of nuclear factor erythroid 2-related factor 2 (Nrf2), a classic antioxidant transcription factor, namely, dimethyl itaconate and 4-octyl itaconate (4OI), decreased IκBζ protein expression in an Nrf2-independent manner, which was related to the regulation of IκBζ translation (42, 43).

IκBζ is also regulated by phosphorylation. IκBζ carries nine phosphorylation sites, including six serine residues and three threonine residues. Two phosphorylation sites (Ser170 and Ser172) are located within the nuclear localization signal. One phosphorylation site (Ser578) is located within the C-terminal ANK domain. Threonine clusters in phosphorylation sites (Thr189/193/195) are highly conserved between species. Notably, the half-life of threonine-phosphorylated and threonine-nonphosphorylated IκBζ did not differ, suggesting that the protein stability of IκBζ is not affected by threonine phosphorylation. However, phosphorylation of IκBζ at Thr189/Thr193/Thr195 promoted the recruitment of histone deacetylase 1 to specific target gene promoters, which resulted in the inhibition of IκBζ target gene expression, such as IL23A, IL6, CXCL5, CCL2 and S100A9 (44). The fusion oncogene FUS-DDIT3 binds directly to IκBζ in a common complex and mediates the nuclear transport of IκBζ (45).

## 5 IκBζ is induced *via* inflammatory signal pathways

**Figure 4:**
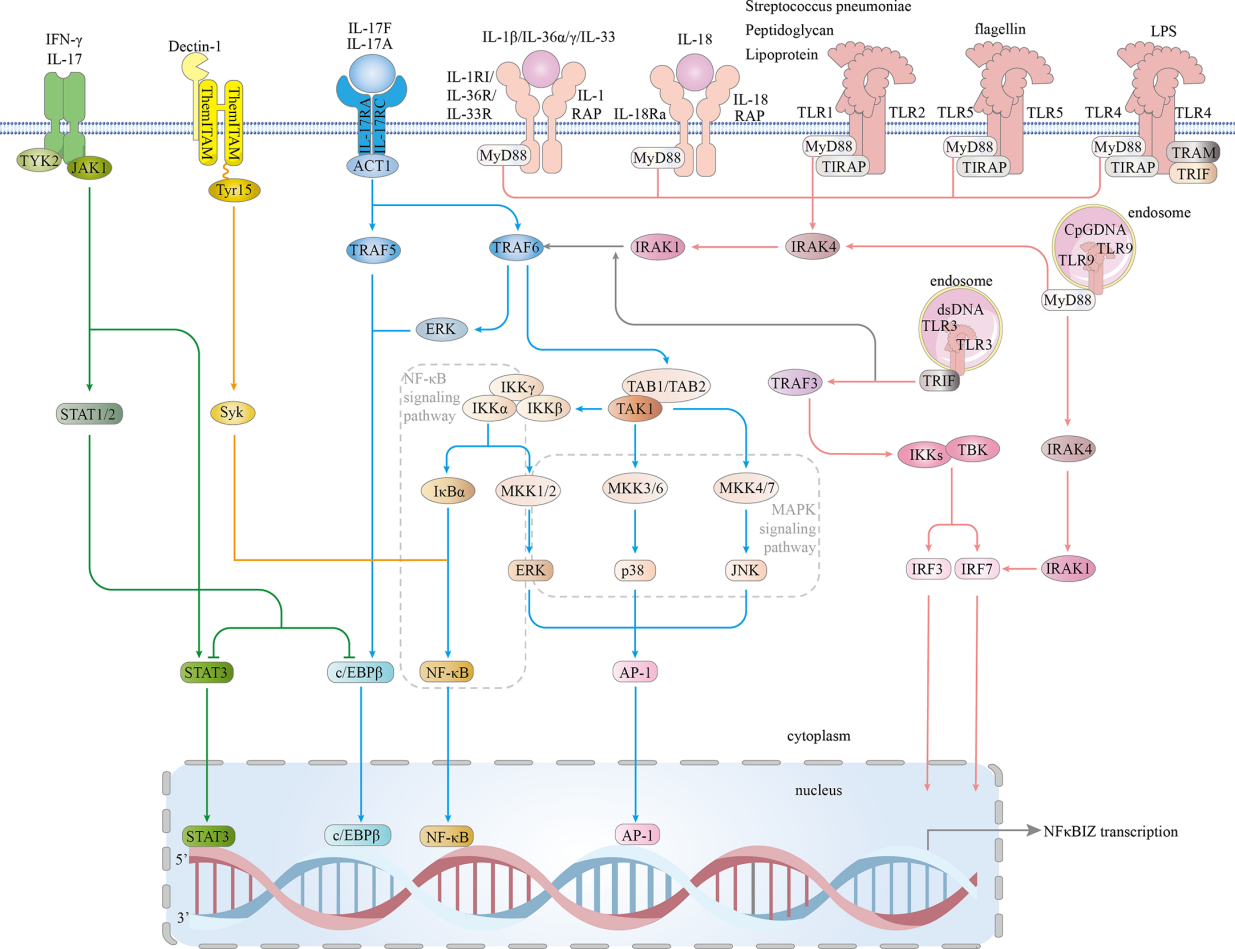
Signaling pathways involved in IκBζ induction. The IL1/TLR-MyD88 pathway: In the IL-1 and TLR (expressed on the cell surface) pathways, signal transduction is activated by peptidoglycan, lipoprotein, Streptococcus pneumoniae, flagellin, or LPS. TLRs expressed on the cell surface (TLR1.2.4.5-complex) then bind to TIR and TIRAP *via* a TIR-TIR domain interaction. IL-1β, IL-18, IL-33, IL-36α and IL-36γ bind to their main receptors, which induces the recruitment of coreceptors. TLRs and the IL-1 and IL-18 receptors are members of the TLR/IL1-R superfamily and share a similar TIR domain in their intracellular region. MyD88 interacts with the TIR domains of TIRAP and TLRs, which then bind to and activate IRAK4/1 and TRAF6. TRAF6 associates with TAK1, leading to the activation of NF-kB, ERK 1/2, p38, and JNK signaling and subsequent translocation of NF-κB and AP-1 into the nucleus, resulting in increased NFKBIZ expression. Intracellular TLRs include TLR3 and TLR9. TLR9 is critical for cellular recognition of CpG DNA. Once activated, TLR9 recruits the adaptor protein MyD88, which subsequently forms a complex with IRAK4, TRAF6, and RAK1. This complex activates NF-kB and phosphorylate IRF7. Then, activated NF-κB and phosphorylated IRF7 are translocated into the nucleus, where they induce NFKBIZ expression. TLR3 is expressed on the endosomal membrane in cells. Uptake of extracellular dsRNA into cells and its subsequent binding to TLR3 leads to TLR3 dimerization, followed by recruitment of the adaptor protein TRIF *via* its transient association with the TIR domain of TLR3. TRIF forms a complex with TRAF3, and together, they recruit and activate TBK1 and IKK, resulting in the phosphorylation of IRF3 and IRF7. Both phosphorylated IRF3 and IRF7 are translocated into the nucleus and induce NFKBIZ expression. IL-17 pathway: In an Act1-dependent mechanism, IL-17A/IL-17F, which combines with the IL-17RA and IL-17RC receptors, recruit Act1, activating TRAF5 and TRAF6, which activate ERK 1/2, p38, JNK, C/EBPβ and NF-kB; NF-kB binds to the nfkbiz promoter. JAK/STAT pathway: JAK1 is activated by IL-17A and IFN-γ. JAK1 phosphorylates STAT3 and STAT1/2. p38 and NF-κB directly bind STAT3. Direct DNA binding by STAT3 triggers IκBζ expression. STAT1 shows the ability to inhibit STAT3- or CEBPβ-induced NFKBIZ promoter activity. Dectin-1 pathway: Tyr15 in the Dectin-1 hemITAM is needed for the subsequent recruitment of Syk, which leads to Syk phosphorylation. Syk induces the expression of NFKBIZ by activating NF-κB signaling. TIRAP-inducing IFN-β (TRIF); Toll/interleukin 1 (IL-1) receptor (TIR); tumor necrosis factor (TNF); receptor-associated factors 3 (TRAF3); TANK-binding kinase 1 (TBK1); IκB kinase (IKK); interferon regulatory factor 3 (IRF3); myeloid differentiation factor 88 (MYD88); IL-1 receptor-associated kinase 4 (IRAK4);receptor-associated factor 6 (TRAF6); IL-1 receptor-associated kinase 1 (IRAK1); interferon regulatory factor 7 (IRF7); Toll/interleukin-1 (IL-1) receptor (TIR); TIR domain-containing adaptor protein (TIRAP); extracellular signal-regulated protein kinase 1/2 (ERK1/2); activator protein-1 (AP-1); Janus kinase 1 (JAK1); signal transducer and activator of transcription 3 (STAT3); signal transducer and activator of transcription 1 (STAT1); spleen tyrosine kinase (Syk); Src-homology 2 (SH2); and hemi-immunoreceptor tyrosine-based activation motif (hemITAM).

On the basis of a recent publication, we classified the inflammatory signaling pathways that induced IκBζ activity into four signaling pathways: MyD88-dependent, IL-17R, JAK – STAT and Dectin-1 pathways. Eventually, the classical MAPK and NF-κB inflammatory signaling pathways are activated, and the STAT3, C/EBPβ, AP-1, and IRF transcription factors are involved in IκBζ induction, which is highly dependent on cytokines and pro-inflammatory bacterial components (**Figure 4**).

### 5.1 MyD88-dependent pathway

One IκBζ production pathway is the MyD88-dependent pathway, which is a part of the IL-1 and Toll-like receptor (TLR) pathways. MyD88 acts as a central molecule in the inflammatory pathway (46). TLRs and interleukin-1 receptor (IL-1R) directly interact with intracellular interleukin receptor-associated kinase (IRAK) family members (47, 48).

Significant IκBζ production is induced by the intraperitoneal injection of LPS (11). Studies have revealed that peptidoglycan, lipoprotein, mycoplasma lipopeptides, flagellin, CpG DNA and double-stranded RNA specifically bind and activate TLRs, thereby inducing IκBζ production (49, 50). IL-1 superfamily members are often classified into four subfamilies based on the coreceptors that they bind: the IL-1, IL-33, and IL-36 subfamilies, whose members function in conjunction with the coreceptor IL-1RAP, and the IL-18 subfamily, which members bind a different coreceptor (51). In IL-1β-treated primary chondrocytes, NFKBIZ is one of the most significantly upregulated genes (39). In IL-36α- and IL-36γ-treated HaCaT cells (keratinocyte cells) and primary human Kupffer cells (KCs), NFKBIZ mRNA and protein expression is increased (19). IL-18 combined with IL-18R induces the expression of IκBζ in KG-1 cells (human acute myeloid leukemia cells) (52). IL-33, a member of the IL-1 cytokine family, binds IL-33R ST2L to activate TLR/IL-1-related signaling factors to induce IκBζ expression (53). *Streptococcus pneumoniae* induces IκBζ expression through the TLR1/TLR2 receptor complex (54).

TLR4 is the predominant form of the LPS receptor and is involved in LPS-induced IκBζ production (55). In humans, TLRs are located on the plasma membrane (TLR1, TLR2, TLR4, TLR5, and TLR6) or in endosomes (TLR3, TLR7, TLR8, and TLR9). Some TLRs undergo homodimerization, and others form heterodimers (e.g., TLR2 binds TLR1 or TLR6) (56). TLRs and receptors of the proinflammatory cytokines IL-1, IL-33, IL-36 and IL-18 share a common Toll/interleukin-1 receptor (TIR) domain in their intracellular region and belong to the TLR/IL1-R superfamily (57–63). Signal transduction is initiated by the activation of receptor domains of TIR after receptors bind of pathogen-associated molecular patterns (PAMPs) and cytokines (59). This activation leads to the recruitment of intracellular TIR-containing adaptors such as MyD88, Mal/TIRAP, TRIF and TRAM (64). MyD88 interacts with IRAKs, including IRAK1, IRAK2, IRAK4 and IRAK-M. The IRAK1/TRAF6 complex dissociates from the receptor and interacts with TGF-β activated kinase 1 (TAK1) and TAK1-binding proteins (TAB1/TAB2), which in turn bind the E3 ligases Ubc13 and Uev1A (65). Once activated, the kinase TAK1 phosphorylates the IKK complex (comprising IKK-α, IKK-β and IKK-γ) and mitogen-activated protein kinases (MAPKs; such as extracellular signal-regulated kinase (ERK) 1/2, C-Jun N-terminal kinase (JNK) and p38) (66). Eventually, the pathway activation cascades reaches the transcription factors NF-κB, AP-1, and IRFs to induce antipathogen responses and inflammation (67–71)

### 5.2 IL-17R pathways

The IL-17 family includes six members (IL-17A, IL-17B, IL-17C, IL-17D, IL-17E, and IL-1F), and IL-17a is generally referred to as IL-17 (72). IL-17A, the most widely studied cytokine in this family, is a pro-inflammatory cytokine (73). Within the larger IL-17 pathway, IL-17R induces IκBζ production (10). IL-17A and IL-1F combine with the IL-17RA/IL-17RC receptors, which subsequently recruit Act1 and activate MAPK and NF-κB (74). IL-17A–induced IκBζ expression is mediated by an Act1-dependent mechanism (75) Moreover, the exogenous IL-17A/IL-1F dimer has been proposed to regulate the expression of IκBζ *via* the involvement of the p38 MAPK signaling pathway (76). TNF-α stimulation alone does not significantly induce the expression of NFKBIZ mRNA, which combines with IL-17A, IL-17F and IL-17A/IL-17AF to increase IκBζ protein expression to high levels. This increase in IκBζ involves the ERK1/2 pathway (10, 76). Notably, PFN1 is an actin-binding protein that inhibits IL-17A-induced IκBζ. (77).

### 5.3 JAK–STAT pathways

The JAK–STAT pathway is involved in almost all immunomodulatory processes and is a common pathway for a variety of cytokines and growth factors (78). IL-17 and IFN-γ activate TYK2 and JAK1 heterodimers. The JAK family comprises four members: JAK1, JAK2, JAK3, and TYK2. The signal transducers and activators of transcription (STAT) proteins are STAT1, STAT2, STAT3, STAT4, STAT5a, STAT5b, and STAT6 (79, 80). Stimulant factors bind to the extracellular domains of the receptor, activating JAK, which in turn phosphorylates STATs. STATs bind directly to DNA and regulate gene expression (81). JAK1 is critical for NFKBIZ expression in the JAK/STAT pathway; IFN-γ together with IL-1 synergistically enhances NFKBIZ expression in both primary keratinocytes, and this process involves JAK1 and NF-κB (82). The expression of NFKBIZ that lacks STAT3- or NF-κB-binding sites is suppressed. Both C/EBPβ and STAT3 are transcription factors that functionally coordinate to induce NFKBIZ promoter activation; moreover, the promoter sequence from positions −219 to −27 in NFKBIZ may play a role in NFKBIZ induction, and STAT1 can counteract C/EBPβ- or STAT3-induced NFKBIZ promoter activity, presumably mediated *via* a coiled-coil-mediated dimer conformation change (83).

### 5.4 Dectin-1 pathways

Dectin-1 is a pattern recognition receptor (PRR) expressed by myeloid cells (macrophages, dendritic cells, and neutrophils) that triggers immediate innate immune responses to fungal and bacterial infections. Dectin-1-mediated signaling stimulates IκBζ production by spleen tyrosine kinase (Syk). Dectin-1 is expressed in mast cells and recognizes β(1→3)-glucan (84). Tyr15 in the hemi-immunoreceptor tyrosine-based activation motif (hemITAM) of Dectin-1 is required for the subsequent recruitment of the Syk, mediated through the SH2 domains of Syk, resulting in the phosphorylation of a Syk tyrosine. In summary, NFKBIZ expression is induced in a Syk-dependent manner (85).

## 6 The inflammatory function of IκBζ

### 6.1 IκBζ regulates the inflammatory cascade response

Transcriptional activation during inflammation is a multistep regulated response. Without the need for *de novo* protein synthesis, primary responses are carried out *via* the rapid activation of NF-κB, which is activated *via* posttranslational modifications (10). In primary responses, IκBζ interacts with inflammatory transcription factors to repress the expression of cytokines, rather than directly activating the transcription of NF-κB target genes suppressed by IκBζ, in ways that are universal and nonselective, and we do not elaborate on them further in this article.NF-κB family members in mammals include RelA/p65, RelB, c-Rel, p50, and p52. They bind as homodimers or heterodimers at κB sites in the DNA of their target genes. NF-kB is involved in the primary immune response by activating transcription factors involved in inflammation. IκBζ binds to NF-κB to downregulate the expression of certain cytokines, such as IL-17, IFN-γ and TNF (86, 87). IκBζ inhibits the DNA-binding activity of p65/p50 heterodimers and p50/p50 homodimers, and it is more likely to be associated with p50 than p65 (6, 14, 52). P50-deficient mice showed responses to TLR/IL-1R ligands that were similar to the responses of sIκBζ-deficient mice (50). Enhanced IgG1 productivity is independent of the p50/p50 activating pathway in NF-κB signaling (88). The alternative transcription of exon 10 results in an IκBζ protein lacking AAs 610–644. These deleted AAs are in an ANK repeat, a domain predicted to interact with the p65/p50 protein (89). In response to IL-17A stimulation, IκBζ protein levels were not significantly reduced by p50 or p65 subunit silencing, decreasing only when both subunits were silenced. Later, studies found that IκBζ interacts with p52 (90). A coimmunoprecipitation assay using an anti-IκBζ antibody revealed that IκBζ interacts with p65, p52 and p50 after stimulation with il-1, and it forms complexes with p65/p50 and p65/p52 heterodimers (17). The subcellular localization of NF-κB subunits and the transcriptional activity of NF-κB were not changed in NFKBIZ^-/-^ keratinocytes (91). Besides, both C/EBPβ and C/EBPδ belong to the CCAAT/enhancer-binding protein (C/EBP) family. IκBζ-mediated activation of the neutrophil gelatinase-associated lipocalin (NGAL) promoter was specifically inhibited in C/EBPβ-depleted cells but not in C/EBPδ-depleted cells. These outcomes illustrate that C/EBPβ is involved in the IκBζ-mediated transcriptional activation of NGAL (92). The interaction between the transcription factor RORγ complex and IκBζ is required for naive CD4(+) T-cell differentiation into Th17 cells (93).

Secondary inflammatory responses are delayed by *de novo* protein synthesis, but they exert a more powerful effect than primary responses. The mRNA level of IL-6 in IκBζ-overexpressing macrophages was higher at the early stage but significantly decreased at the later stage in the LPS response (94). In response to IL-36α stimulation, IκBζ downregulated the expression of genes for anti-inflammatory phosphatases (DUSP2 and DUSP9) after 1.5 h, and after 24 h, the expression of most of the genes regulated by IκBζ increased (19). This evidence indicates that IκBζ is temporally specific for the expression of target genes. IκBζ binds to NF-κB to suppress the expression of NF-κB target genes in the primary inflammatory response but promotes the expression of target genes in the secondary response. These secondary response genes targeted by IκBζ are recognized as IκBζ target genes (**Table 2**). Target gene expression is collaboratively induced by IκBζ interacting with NF-κB, especially the p50 subunit (15, 100).

**Table 2:** Sort out the IκBζ activity mediated by different stimulus.

Stimulus	Target genes	Activity	References	Other site in the DNA of target gene. (IκB-ζ relate to other site)	Other binding transcription factor	Cell types
IL-12/IL-18	IFN-γ	increase	(86)	C3-3P(NF-κB binding sites)	NF-κB	HEK293 cells
LPS	IL-6	increase	(54, 94, 95)	NF-κB binding sites	NF-κB	HEK293 cells, Bone marrow-derived macrophages, murine bone marrow-derived dendritic cells
GPI	IL-12b	increase	(96)	NF-κB binding sites	NF-κB	Macrophages
/	LCN2	increase	(97)	GGGAATGTCCC at positions –230/–220 from transcription start site(NF-κB binding sites)	p50 (NF-κB)	/
LPS	IL-10	increase	(98)	NF-κB binding sites	p50 (NF-κB)	RAW264.7 cells
LPS	CCL2	increase	(99)	NF-κB binding sites	NF-κB	RAW264.7 cells
LPS	TNF-α	Inhibit	(100)	NF-κB binding sites	p50 (NF-κB)	RAW264.7 cells
IL-17A/F	CCL20, DEFB4, IL-8, CHI3L1, S100A7	increase	(76)	/	/	human keratinocytes
IL-12	IFN-γ	Increase	(101)	/	/	natural killer cells
D39	GMCSF	Increase	(54)	/	/	HEK293 cells
Rv1759c	miR-25	Increase	(102)	/	/	Mycobacteriumtuberculosis (Mtb)
/	COL1A2	inhibit	(103)	/	/	/
IL-36γ	IL-23	increase	(104)	/	/	murine bone marrow-derived DCs (BMDCs)
IL-17A	NOS2	Increase	(105)	/	/	human ulcerative colitis epithelium

### 6.2 IκBζ regulates the expression of cytokines by participating in chromatin remodeling

Nucleosome remodeling participates in chromatin remodeling by altering the interaction activity of DNA with histones, including switch/sucrose-nonfermentable (SWI/SNF) complexes. Akirin2 is a protein with conserved sequences that regulates gene expression. The ANK domain of IκBζ interacts with the conserved C-terminal region of Akirin2. IκBζ–Akirin2 binds to SWI/SNF complexes, which are involved in the induction of a set of genes, such as IL-6, in response to IL-1β/TLR stimulation (95).

Histone modifications are also influential in chromosome remodeling. H3K4me3 (H3K4 trimethylation) is a histone mark indicating open chromatin, and it is linked to promoter regions of active transcriptional genes. IκBζ inhibited dentin extracellular matrix (ECM)- and ECM organization-related gene expression through altered local chromatin that is marked by H3K4me3 (103). IκBζ results in abundant H3K4 trimethylation at the CCL2, Lcn2, IL-10 and IL-6 promoters but not the Cxcl2 promoter (98, 99, 106). Details explaining the mechanisms underlying the association of IκBζ with H3K4 trimethylation remain to be determined.

## 7 IκBζ in immune cells

### 7.1 Mononuclear phagocyte system

IκBζ mediates synovial fibroblast recruitment of neutrophils and monocytes (87). Early in Salmonella infection, IκBζ is involved in the suppression of macrophage action with respect to Salmonella (107). Macrophages have been extensively studied as canonical cellular models in mechanistic studies of IκBζ action. Oxidative stress is associated with the LPS-induced upregulation of IκBζ in macrophages (108). Peritoneal macrophages predominantly express the splicing variant IκBζ(L) (57). Cardiac macrophages derived early in development show increased NFKBIZ expression. (109) Intron retention (IR) is one mode of alternative splicing that occurs when an intron is not excised and is thus preserved within mature mRNA (110, 111). NFKBIZ expression is regulated by IR during macrophage activation, and NFKBIZ transcripts are retained in the nucleus of macrophages before activation, enabling macrophages to rapidly express key inflammatory regulators (112). IκBζ-deficient macrophages exhibited impaired secretion of CCL2 (99). LPS-induced GM-CSF and G-CSF expression in peritoneal macrophages greatly depends on IκBζ (50) (**Table 2**). R848 (a TLR8 agonist)-treated neutrophils continued to induce IκBζ protein expression for 1 h, with the level peaking at 3 h and remaining stable for up to 20 h, while R848-stimulated monocytes showed short-lived IκBζ protein expression (113).

### 7.2 NK cells

IκBζ is essential for the activation of NK cells and antiviral host defense responses; however, it is dispensable in the differentiation of NK cells. IκBζ is essential for IL-12 and IL-18 activation in NK cells and promotes the expression of IFN-γ. Recruitment of IκBζ to the IFN-proximal promoter region promotes transcriptional activation, which is a hallmark of NK-cell activation (86).

### 7.3 T cells

IκBζ-deficient naive T cells cannot differentiate into T helper 17 (Th17) cells because the differentiation of TH17 cells requires the interaction between the transcription factor RORγ complex and IκBζ (93). IκBζ-deficient T cells exhibit reduced immunoregulatory function and are associated with mouse immune response dysregulation; moreover, IκBζ is not necessary for regulatory T cell (Treg) plasticity or stability (114).

### 7.4 B cells

Costimulation of a BCR and TLR9 or TLR7 synergistically induces IκBζ expression in B cells. BCR-mediated induction of IκBζ by PI3K does not depend on TLR; however, the expression of FcγRIIB, CD72 and CD22 was reduced and induction of IL-10 mRNA was abolished in IκBζ-deficient B cells following TLR9 stimulation (115). Stable B-cell lines overexpressing NFKBIZ showed a specific 1.2-1.4-fold increase in IgG1 production 1 (88).

## 8 IκBζ knockout is associated with proinflammatory properties

*NFKBIZ^-/-^* mice develop spontaneous skin inflammation that can progress to Sjögren’s syndrome and exhibit dry eyes and mouths, indicating the occurrence of inflammation on the ocular surface and perioral skin, resulting from inflammation and apoptosis (116–118). IκBζ-deficient epithelial cells in the lacrimal gland exhibit accelerated apoptosis (119, 120). *NFKBIZ^-/-^* keratinocytes are hypoproliferating cells(Ishiguro-Oonuma et al., 2015). Knocking down IκBζ expression induces toxicity in activated B-cell-like subtype of diffuse large B-cell lymphoma (ABC DLBCL) cell lines (90). IκBζ-knockdown HaCaT cells (keratinocyte cells) exhibited abrogated IL-36-mediated upregulation of multiple genes associated with psoriasis (19).

## 9 Diseases related to IκBζ in the context of inflammation

### 9.1 Psoriasis

Psoriasis is the most frequently mentioned chronic inflammatory disease in the NFKBIZ-related literature. Psoriasis is a multifactorial disease with genetic factors accounting for approximately 70% of the disease susceptibility (121). IκBζ plays a crucial role in the development of psoriasis (122). IκBζ is a direct transcription activator of psoriasis-related genes; the single-nucleotide polymorphism (SNP) rs7637230 in NFKBIZ (position 101663555) is related to psoriasis mobility, and an adenine in this SNP increases the risk of developing psoriasis compared with the risk associated with guanine (OR=1.14) in individuals of European descent (123). Significant differences in the frequency of this NFKBIZ polymorphism (intron 10 indel) were observed between Cw6-positive and Cw6-negative psoriasis patients (89). HLA-Cw6 is among the alleles most closely associated with psoriasis susceptibility (124). IκBζ-deficient mice showed inhibited development of IL-17-, IL-23- and imiquimod-induced psoriasis, and IκBζ-deficient keratinocytes attenuated IL-36–induced psoriasis-like dermatitis (125, 126). IL-36-mediated IκBζ and secondary inflammatory gene expression, e.g., DEFB4, CCL20, S100A7 and LCN2 gene expression, following kinetics that were similar to those observed after IL-17A/TNFα treatment in HaCaT cells (keratinocyte cells) and in primary keratinocytes (19, 76).

### 9.2 Cancer

There is a close relationship between inflammation and cancer, and inflammation plays a decisive role in different stages of tumor development, including initiation, promotion, malignant transformation, invasion and metastasis (127, 128). The tumor microenvironment consists of innate and adaptive immune cells that interact with cancer cells through direct contact or the production of cytokines and chemokines (129). IκBζ plays central roles in regulating cytokines and their expression in various immune cells and regulating the differentiation of immune cells, indicating that IκBζ may play a role in the tumor microenvironment (114). Both the coding region and the 3′-UTR in NFKBIZ can drive cancer genesis and progression (130). NFKBIZ appears to serve as an oncogene in hematologic tumors. The expression of *NFKBIZ* was increased in ABC DLBCL and primary testicular diffuse large B-cell lymphoma (PT-DLBCL) contexts, specifically acting as a promoting factor in DLBCL. (131) Somatic *NFKBIZ* is mutated in PT-DLBCL, and approximately 40% of PT-DLBCL cases investigated in a study were associated with increased *NFKBIZ* expression (131, 132). The oncogenic RAS transcriptome is regulated by IκBζ, the deficiency of which reduces RAS-mediated tumorigenesis-related cytokine expression; IκBζ thus may be a prognostic marker of cancer progression (113). One of the characteristic lesions in ABC DLBCL is associated with a 1.08-Mb amplicon on chromosome 3, which is found in 9% of cases. This amplification segment includes NFKBIZ loci (133). IκBζ activity was markedly induced by CpG (a TLR ligand) in chronic lymphocytic leukemia (CLL) cells, and IκBζ controlled an oncogenic pathway relevant to mature B-cell neoplasia (134). NFKBIZ-mutant lymphomas are mutation-driven malignancies that are associated with a greater number of activated B-cells (135).

The reported effects of NFKBIZ on solid tumors have been inconsistent. NFKBIZ is considered an oncogene in colorectal cancer (CRC). Specifically, NFKBIZ has been identified as a novel potential CRC susceptibility gene by exome sequencing of 29 samples from 43 CRC patients that showed familial clustering (136). The downregulation of *NFKBIZ* was observed in tumor tissue compared to that in paired healthy mucosa from CRC patients (137). NFKBIZ mutations are very common in the epithelium of patients with ulcerative colitis but are rarely found in sporadic or colitis-related cancers, suggesting the selection of NFKBIZ-mutant cells during colorectal carcinogenesis (138). NFKBIZ is a highly mutated genes in rectal cancer, and mutations in NFKBIZ have been associated with chemoradiotherapy (CRT) resistance (139). However, some evidence has suggested that NFKBIZ acts as a tumor suppressor gene in bladder cancer (BC) and lung adenocarcinoma (LUAD). Specifically, NFKBIZ expression was significantly downregulated in BC, and these BC patients exhibit a poor prognosis because NFKBIZ inhibited BC cell proliferation through the PTEN/PI3K/Akt signaling pathway (140). NFKBIZ mRNA was undetectable in unstimulated renal cell carcinoma cells (141). Low levels of NFKBIZ expression in LUAD patients have been associated with reduced overall survival (142). DLG2 is an important tumor suppressor gene, and DLG2 overexpression leads to an increase in the expression of IκBζ (137).

### 9.3 Other diseases

In addition, NFKBIZ upregulation has been reported in infection, kidney injury, enteritis, autoimmune disorders, metabolic diseases, and hypoxic damage. Infectious diseases: NFKBIZ was found to be highly correlated to mastitis in a gene coexpression network analysis (WGCNA) (143). Kidney injury: NFKBIZ is a hub gene in acute kidney injury (AKI) according to a bioinformatics-based study (144). Enteritis: IL-17 significantly promotes the expression of NFKBIZ in intestinal fibroblasts from Crohn’s disease (CD) patients (145). By causing dysbiosis and the obvious growth of segmented filamentous bacteria, loss of IκBζ in intestinal epithelial cells enhanced the development of Th17 cells and exacerbated inflammatory disorders in mice. IκBζ deficiency caused Paneth cell numbers to decline and IL-17-inducible genes that are involved in IgA synthesis to be expressed less efficiently. The loss of Paneth cell integrity and reduced IgA secretion are critical for microbiota dysregulation (146). Autoimmune disorders: NFKBIZ expression was found to be upregulated in both T cells and B cells on the basis of scRNA-seq data obtained from chronic antibody-mediated rejection (cABMR) patients (147). Neuronal ischemia: The lncRNA TCONS_00041002 may inhibit apoptosis and promote neuronal activity by upregulating the expression of NFKBIZ, attenuating hypoxic-ischemic encephalopathy (148). In inflamed tissues of patients with ulcerative colitis, the mutation rate of NFKBIZ was significantly higher than that of PIGR, TRAF3IP2, TP53, ADID1A, or ZC3H12A (105). IL-1β induced IκBζ production in bronchial epithelial cells, and IκBζ mediated asthma-related inflammation in lung epithelium (16). Reduced expression of NFKBIZ reduced renal injury in patients with septic AKI *via* the regulation of miR-376b action (26). NFKBIZ expression was increased in porcine alveolar macrophage (PAM) infection and porcine reproductive and respiratory syndrome virus (PRRSV) infection (149).

NKFBIZ polymorphism associated with diseases: NFKBIZ rs3217713 is a 23-nt insertion/deletion (indel) polymorphism, and the deletion allele is more common to coronary artery disease patients than in other Caucasians from the Asturias region (150, 151). NFKBIZ rs645781 and NFKBIZ rs677011 have been closely associated with the risk of invasive pneumococcal disease (IPD) (152). Lipin1 coordinates liver metabolism, and IκBζ ameliorates fatty liver by downregulating Lipin1 (153). The NFKBIZ rs3217713 II genotype has been associated with a higher risk of death among COVID-19 patients with severe infection in the ICU (154). Hidradenitis suppurativa patients have been shown to carry the NFKBIZ homozygotic variant SNP rs3217713 (155).

## 10 Discussion

The effect of IκBζ on autoimmune diseases has been widely studied, and among these diseases psoriasis is the most studied autoimmune disease. IκBζ is upregulated in these diseases. IκBζ may be used as one of the diagnostic markers of autoimmune diseases. IκBζ is considered to be a target in the treatment of psoriasis, Psoriasis and IRI-AKcan be treated by inhibiting the expression of IκBζ. The mechanism underlying psoriasis treatment mediated by narrowband ultraviolet B (NB-UVB) light has been associated with the decreased expression of NFKBIZ expression (156); however, this treatment target has been validated only in animal models. The delivery of *NFKBIZ* siRNA *via* ionic liquid inhibited imiquimod-induced psoriasis in mice (157). Silencing IκBζ by exposing it to short interfering RNA (siRNA) decreases the IL-17-induced expression of psoriasis-related genes, including CCL20, DEFB4, IL-8, CHI3L1 and S100A7 (76). There is some evidence that IκBζ can serve as a treatment target in human. Adalimumab, an anti-TNF antibody, and secukinumab, an anti-IL-17A antibody, have been approved for psoriasis treatment. NFKBIZ induces psoriasis-related gene expression, which was significantly decreased after 4 days of secukinumab treatment (75). The NFKBIZ polymorphism (rs3217713) has been associated with the adalimumab response, and NFKBIZ-deletion-allele carriers are found at a significantly greater frequency among responders to adalimumab in the Caucasian population with psoriasis than among nonresponders (158).

IκBζ is indispensable for the function and differentiation of immune cells. Immune cells play an important role in the occurrence and development of tumors. The function of IκBζ varies with the type of cancer. It is suggested that the role of IκBζ in tumors is complex. In addition, IκBζ plays an important role in tumorigenesis and development, and it can be used as a prognostic marker of cancer progression. We summarized the upstream signals of IκBζ, but mostly in the inflammatory background. The oral administration of the flavonoid apigenin reduces the elevation of IκBζ mRNA levels in the kidneys of aged rats, which is beneficial for the treatment of late-stage cancer (159). Therefore, the expression level of IκBζ in various types of cancer may require further study to explain the upstream signal of IκBζ, especially since the inhibitory signal of IκBζ may be used as a target for the treatment of corresponding cancers.

## Author contributions

All authors listed have made a substantial, direct and intellectual contribution to the work, and approved it for publication.
