# Corrigendum: The central inflammatory regulator IκBζ: induction regulation and physiological functions

**DOI:** 10.3389/fimmu.2024.1398222

**Published:** 2024-04-08

**Authors:** Yanpeng Feng, Zhiyuan Chen, Yi Xu, Yuxuan Han, Xiujuan Jia, Zixuan Wang, Nannan Zhang, Wenjing Lv

**Affiliations:** ^1^ Department of Neurosurgery & Pathophysiology, Institute of Neuroregeneration & Neurorehabilitation, Qingdao University, Qingdao, China; ^2^ Department of Geriatrics, The Affiliated Hospital of Qingdao University, Qingdao, China

**Keywords:** IκBζ, inflammation, tumor, psoriasis, toll-like receptors, mRNA

**Error in Author List**

In the published article, there was an error in the author list, and author **Zhiyuan Chen** was erroneously included as a co-first authors. The corrected author list appears below.

Yanpeng Feng^1^,Zhiyuan Chen^1^,Yi Xu^1^,Yuxuan Han^1^,Xiujuan Jia^2^, Zixuan Wang^2^,Nannan Zhang^2^,Wenjing Lv^1,2*^

The authors apologize for this error and state that this does not change the scientific conclusions of the article in any way. The original article has been updated.

